# Reproductive Healthcare Needs of Sex Workers in Rural South Africa: A Community Assessment

**DOI:** 10.5334/aogh.2706

**Published:** 2020-06-29

**Authors:** Omara Afzal, Molly Lieber, Ann Marie Beddoe

**Affiliations:** 1 Department of Obstetrics and Gynecology, Icahn School of Medicine at Mount Sinai, New York, US

## Abstract

**Background:**

In the Limpopo province of South Africa, access and availability of women’s health services are limited and many challenges exist for a growing population of transient sex workers. This study was developed to place communities at the forefront to more specifically understand regional barriers and attitudes regarding reproductive health care needs.

**Objective:**

To build strong community partnerships, gain understanding of issues in women’s health services, and collaborate with community members to address those issues.

**Methods:**

A mixed-methods study approach was used in rural South Africa. Participants were recruited through voluntary interest from a local health clinic performing outreach for migrant female sex workers. We (1) created partnerships and built trust within the community and (2) worked collaboratively to collect both qualitative and quantitative data, using community groups to discuss health needs as well as “knowledge, attitude, and practice” (KAP) surveys.

**Findings:**

Ninety-four sex workers participated. The survey data identified risk factors to poor reproductive healthcare outcomes, including limited education, young age at first sexual contact, large number of sexual partners, little knowledge of sexually transmitted infections, distrust in the use of healthcare facilities, and limited use of contraception. Community discussion groups revealed a desire for easier and more accessible healthcare, showing the biggest barriers to care are lack of money and transportation, and safety concerns related to profession, including fear of violence from partner and/or client. With input from civic leaders, public interest, and community outreach groups, a community advisory board was successfully formed for future collaboration.

**Conclusion:**

By working with local stakeholders and sex workers, we created an interactive and tailored assessment to discuss healthcare disparities. We helped foster community ownership, setting the stage for future implementation of sustainable and cooperative health programming.

## Introduction

The Limpopo province of South Africa is one of the poorest in the country. It has a population of over 5 million people, 80% percent of whom live below the poverty line [1]. Employment, one of the key determinants to community empowerment, is difficult to obtain. Thus, farm work and sex work have become important sources of economic survival, particularly for women in this region. Of these two groups, female sex workers (FSW) are particularly vulnerable and are at high risk for sexually transmitted infections (STIs) and other medical and social comorbidities. A largely ignored and marginalized population, the reproductive health needs of FSWs have been minimally assessed and mostly unmet [2]. Due to the criminalization of the profession, stigma, and violence, there is often limited access and availability to health services for FSWs, likely contributing to the high prevalence rate of HIV in this population [2]. In sub-Saharan Africa, up to 4.3% of women and girls engage in sex work; an estimated 37% of FSWs are infected with HIV, 60% are unaware of their status, and the odds of HIV infection among FSWs is 13.5 times higher than that of other women [3,4]. There are multiple factors placing these women at increased risk of STIs, including multiple sex partners, unprotected intercourse, and unsafe environments leading to further adverse sexual and reproductive health outcomes [4]. In South Africa, there is little information on the numbers, characteristics, behaviors, and health needs of FSWs, though estimations for HIV in varying FSW communities range from 45% to 60%, and it is estimated that approximately 20% of new HIV infections involve sex work, signifying a need for health attention [5,6].

In Hoedspruit, an agricultural and game farming community in the Limpopo province, the estimated prevalence of HIV infection among local sex workers is 34% to 69%, with many women remaining untested [1]. A local HIV wellness clinic in this region has reached out to the FSW community with the establishment of health care advocate programs to increase awareness, prevention, and treatment of HIV among this high risk population. Additionally, they have taken on the task of education and community engagement of FSWs in the region through a peer education program with fellow and former sex workers. While it has been very successful, there is still an overwhelming need for a program in the Limpopo province to address the more complex sexual and reproductive health care needs of this growing regional population as accessibility and availability of health services, such as preventative care, screening, and contraception, are limited. A public health campaign with community involvement could prospectively alter the rates of morbidity and mortality associated with poor reproductive health outcomes. This study was developed to place communities at the forefront to more specifically adapt a public health response to the region’s barriers and attitudes. The objective was to build strong partnerships and to gain a better understanding of the existing gaps in reproductive health services available to FSWs and to collaborate with community members to address those unmet needs.

## Methods

Approval was obtained from the Institutional Review Board at the Icahn School of Medicine at Mount Sinai in New York, USA, and the Faculty of Health Sciences Research Ethics Committee at the University of Pretoria, South Africa, to conduct this mixed-method study. Community involvement and buy-in, both crucial components of this project, were achieved through (1) creating partnerships and building trust within the community and (2) working together with the community to design surveys and collect both qualitative and quantitative data. Through an iterative and linguistic process, and in collaboration with local nurses and medical assistants who work with this population, a survey was designed and pilot tested through three revisions based on feedback to ensure the final instrument was culturally appropriate and sensitive. The knowledge, attitudes, and practice (KAP) survey consisted of 32 questions assessing the following: basic demographics (age, education level, country of origin, migrant status, current residence, religion, and occupation), reproductive health care/basic obstetrical care (age at first intercourse, number of sexual partners, number of children, history of abortion, how and where performed, and smoking status), family planning (use of contraception, types used in the past and currently, understanding of condom use and purposes), sexually transmitted infections (knowledge of types of STIs, history of infection, place of treatment, knowledge of HPV and cervical cancer, partner circumcisions, risk factors, and protection from cervical cancer), and gender-based violence experiences (history of, by whom, reporting to authorities, and seeking medical care).

Self-identified sex workers who presented to a community outreach and wellness clinic at different times in 2016 and 2017 in Hoedspruit, South Africa, were offered the opportunity to participate in the surveys, interviews, and group discussions. Recruitment was conducted through word of mouth and at the time patients presented for their routine care at the clinic. No incentive was given to participate, and informed consent was obtained from all participants. Surveys were conducted by clinic staff and volunteers who were not directly involved in patient care and who had undergone training in sensitivity and cultural competence, as well as ethics in health care, by research investigators prior to beginning the study. Questions were read to the patient and answers were recorded. Additionally, informal interviews were conducted following the survey with participants who wished to provide further insight and in-depth information regarding their sexual and reproductive health care challenges. To further ascertain personal experiences and perspectives of the sex worker participants, three focus groups with five to seven participants each were conducted and facilitated by local health workers using open-ended questions regarding the status of the population in matters of reproductive health care issues in the community.

Information collected from the survey questionnaires, interviews, and focus and discussion groups were compiled. Quantitative data was analyzed to obtain demographic data on the population and social and behavioral risk factors, as well as knowledge deficits on key reproductive health issues. Qualitative data was assessed for common themes, repeated phrases, and areas of high interest as perceived by the participants themselves. Identification of perceived needs by sex workers were highlighted for future programs involving discussion, lectures, and outreach on specific topic areas. Community leaders, advocates, health care providers, participants themselves and other stakeholders were included in key issue discussions for future programming, which is not described in this study, including topics such as sexually transmitted infections, risk factors, and treatment options, as well as gender-based violence concerns.

## Results

Ninety-four sex workers were surveyed and participated in qualitative discussion groups. Participants ranged in age from 18 to 44, with the average age of 27.59 years. Thirty-four percent of FSWs reported they were HIV-positive (n = 32); whereas, the remaining 66.0% were self-reported negative or status unknown (n = 62) (Table 1).

**Table 1:** Baseline characteristics of female sex workers in Limpopo Province, South Africa (N = 94).

	N = 94 n (%)

**Mean age in years +/– SD**	27.6 +/– 0.6
**Mean age in years at first coitus +/– SD**	16.7 +/– 1.7
**Education**	
No school	6 (6.4)
Some primary school	18 (19.1)
Some high school	52 (55.3)
Completed high school	18 (19.1)
College or more	0 (0)
**Status**	
HIV positive	32 (34.0)
HIV status unknown/self-reported negative	62 (66.0)
**Sexual Partners**	
1–4	0 (0)
5–9	11 (11.7)
10–20	10 (10.6)
>20	46 (48.9)
Do not know	24 (25.5)
No answer	3 (3.2)
**Contraception Use**	
None	13 (13.8)
Condom use	75 (79.8)
Other, no condom	6 (6.4)

SD – standard deviation.

### Quantitative

Survey data identified multiple risk factors and barriers to reproductive healthcare for a majority of responders, including limited education, young age at first coitus, multiple sexual partners, lack of sexually transmitted infections knowledge, distrust in healthcare facilities, and limited use of contraception. Approximately 81% of sex workers had not completed high school (n = 76), 48.9% of women reported more than 20 sexual partners (n = 46), with 25.5% of women responding they did not know how many sexual partners they have had (n = 24), and almost 80% of women reported use of condoms most of the time but all reported little use of other forms of contraception. Demographics and contraception use are included in Table 1. As a result of counseling received at the HIV wellness center, all but four women were aware of HIV but had little knowledge of other STIs and had no understanding of HPV or cervical cancer risk factors or symptoms (Table 2). Approximately 45% of participants reported being victims of gender-based violence (GBV) (n = 42), with a 9.6% no-response rate (n = 9). Of those who responded, 83.3% did not report to the authorities or seek medical care (n = 35). Reported perpetrators of violence were largely partners and clients.

**Table 2:** Awareness of specific Sexually Transmitted Infections among Female Sex Workers.

Variable	HIV (*n* = 94)	Chlamydia (*n* = 94)	Gonorrhea (*n* = 94)	Syphilis (*n* = 94)	Hepatitis (*n* = 94)	HPV (*n* = 94)	CC risk* (*n* = 94)

Knowledge, n (%)	90 (95.7)	4 (4.3)	8 (8.5)	4 (4.3)	1 (1.1)	3 (3.2)	3 (3.2)
No Knowledge, n (%)	4 (4.3)	90 (95.7)	86 (91.5)	90 (95.7)	93 (98.9)	91 (96.8)	91 (96.8)

* CC – Cervical cancer risk from HPV.

### Qualitative

Among many revelations from community discussion groups, a desire for easier and more accessible healthcare was at the forefront. Barriers to care included lack of finances and accessible transportation, as well as safety concerns related to profession, including fear of violence from partner and/or client. Table 3 outlines common themes expressed by participants during focus group discussions on the largest concerns of individuals and how to improve the FSW community as perceived by participants.

**Table 3:** Focus Groups: Most Commonly Expressed Themes.


**Biggest Concerns of FSWs**	“I am afraid of police, but not so much anymore because the police also buy us.”
	“We worry we will not get the money that was agreed on before we start.”
	“I am in a situation that forces me to do this…we need the money.”
	“There are men who will hurt you…sometimes they will be drunk and abusive. [Many men] do not want to use condoms.”
	Worried about her children being supported if she is hurt
	Diseases through sex
	Pressure of friends and housemates to do this job for money
**How to Improve the FSW Community**	“We need jobs. I want a job so I can stop selling my body. [Some of us] don’t want to work as a sex worker anymore”
	“There should be training for jobs. There are no jobs for someone like me here”
	“I want to go back to school…maybe [then] I would not have to do this.”
	“The government should protect sex workers…they can help us a specific place to stay and we can be safe with a salary each month.”
	Safe and protected place to perform the job
	Regular health care to check for diseases
	Group meetings and working together for a support system


## Discussion

Our results indicate that in this marginalized community multiple risk factors and barriers to reproductive health care exist. Lack of education and knowledge of health issues seemed most influential on sexual and reproductive health behaviors and outcomes, with STIs being a prevalent concern but with little understanding of the infections. There was also a general feeling of disempowerment expressed in discussion groups, with a sense there were no alternative options for employment and limited community support.

Community empowerment among FSWs is a process necessary for the accessibility and acceptability of health care services for the population [3]. The access and use of women’s health services is known to be limited overall in this population. A brothel-based clinic in Hillbrow, South Africa, was found to have a positive influence on health-seeking behaviors and awareness, including condom use, showing success with innovative and community involved programs [5]. Programming that involves the community it targets are possibilities for future directions in this area. Two highlighted issues among FSWs in our study group were knowledge and prevention/treatment of STIs and fear of GBV. Sex workers are at a higher risk of experiencing GBV from clients, controllers, law-enforcement, family, and intimate partners, among others, and many of the participants in this study expressed concern and fear regarding GBV [7]. A comprehensive approach to prevent and respond to GBV can include awareness of current protective policies and law, training of law-enforcement, and creation of programs to promote economic security [8]. Financial deprivation is a significant social factor contributing to sex work and a motivation to remain in the profession. Programs to address economic strain may aid community needs [9].

A public health campaign with active community involvement in a mutually respectful, non-judgmental manner has the potential to alter the rates of morbidity and mortality associated with early or unintended pregnancy, sexually transmitted infections, GBV, and cervical cancer rates. There is a high prevalence of cervical pre-cancer and cancer among FSWs, yet screening is rarely performed [4]. In addition to difficulties in accessing health care, stigma and poor quality of care limit FSWs’ willingness to seek care [10]. Effective partnerships and collaborations between key stakeholders, including sex workers themselves, community leaders, and healthcare providers, among others, is necessary for building community capacity and designing acceptable intervention strategies [2]. Assessing reproductive health needs through the community allows for receptive and responsive programs to *expressed* needs rather than outside *perceived* issues. Further, training health care workers in stigma-reducing sensitization and support for FSW health may improve care in the community and identify other sources of vulnerability, including increased assessment of high-risk issues [4,8,11].

Limitations of this study include the small sample size and the possibility of selection bias given participants’ previous involvement in HIV community outreach within the HIV clinic. Additionally, though clinic personnel and volunteers underwent training in survey administration and cultural competency, there is a possibility of both moderator bias and respondent bias due to fear of judgement. Strengths of this study include the trust and partnerships formed among the community leaders and sex worker population themselves to initiate assessment and action. Much research on this marginalized population fails to accurately delve into sex worker experiences and expressed needs, and publicly, there is a lack of awareness and concern for their reproductive health care challenges [9]. Forming collaborations with community leaders is the first step in addressing the needs of this marginalized population and reducing health disparities within the community. With input from civic leaders, public interest, and community outreach groups, we successfully established partnership with the community advisory board for future collaboration. This partnership involves sex workers, local health organizations, community leaders, secondary stakeholders, and researchers. Additionally, we heightened awareness of the importance of reproductive health and highlighted barriers so future research can be adapted to local community needs.

An improvement in the use of reproductive health services in the region has the potential to affect pregnancy rate, sexually transmitted infections, and adverse maternal outcomes, as well as improve overall health status and education. Once a campaign is instituted, it can be continued through community partnerships and maintained at a local level. A successful program will lend itself as a model for nearby communities with similar barriers to reproductive health care and can possibly be implemented on a much larger, national scale. By working with local stakeholders and sex workers, we created an interactive and tailored assessment to discuss healthcare disparities. We helped foster community ownership of local healthcare provision, contributing to the efficacy and sustainability of future projects. We demonstrated successful implementation of both qualitative and quantitative research, setting the stage for future research in which partners will create and implement a sustainable and cooperative public health campaign. Future projects will include further analyzing responses, creating tailored interventions, and implementing projects with input and guidance of the community advisory board.

## Conclusion

HIV programs globally have identified FSWs as a high-risk population, often focusing on condom distribution and HIV prevention interventions. However, stigma and criminalization of sex work may create a direct barrier to accessing HIV health care and to prevention [3]. Sex workers report humiliation and fear of refusal of service and other negative experiences that may preclude seeking care from public health services [5]. Additionally, few programs address other health issues, including counseling or termination of pregnancy services, cervical cancer screening, or gender-based violence support; and though some include peer education projects, it is often entirely with non-sex worker personnel [3]. The most effective prevention strategies for sexual and reproductive health services have involved community-based programs, with meaningful participation of FSW communities in program design and implementation [10]. Much research on FSW in Africa has focused on the potential for spreading infection rather than the self-expressed needs of these women [11]. Self-advocacy through assessment and action can improve education and health care on issues important to the community themselves, for which they may not have been otherwise reached through mainstream services [10,11] Community empowerment through self-identified needs can help create sustainable improvements in reproductive health care to a high-risk population.
